# Enoxacin and bis-enoxacin stimulate 4T1 murine breast cancer cells to release extracellular vesicles that inhibit osteoclastogenesis

**DOI:** 10.1038/s41598-018-34698-9

**Published:** 2018-11-01

**Authors:** Taylor C. Vracar, Jian Zuo, JeongSu Park, Demyana Azer, Christy Mikhael, Sophia A. Holliday, Dontreyl Holsey, Guanghong Han, Lindsay VonMoss, John K. Neubert, Wellington J. Rody, Edward K. L. Chan, L. Shannon Holliday

**Affiliations:** 1 Department of Orthodontics, University of Florida College of Dentistry, Gainesville, FL 32610 USA; 2 Department of Oral Geriatrics, School and Hospital of Stomatology, Jilin University, Changchun, 130021 P. R. China; 3 Department of Oral Biology, University of Florida College of Dentistry, Gainesville, FL 32610 USA; 4 Department of Anatomy and Cell Biology, University of Florida College of Medicine, Gainesville, FL 32610 USA

## Abstract

Enoxacin and its bone-seeking bisphosphonate derivative, bis-enoxacin, have recently captured attention as potential therapeutic agents for the treatment of cancer and bone disease. No differences in growth or survival of 4T1 murine breast cancer cells were detected at a concentration of 50 µM of enoxacin or bis-enoxacin. Growth was perturbed at higher concentrations. Both 50 µM enoxacin and bis-enoxacin stimulated increases in the number of GW/Processing bodies, but there were minimal changes in microRNA levels. Extracellular vesicles (EVs) released from 4T1 cells treated with 50 µM enoxacin or 50 µM bis-enoxacin stimulated proliferation of RAW 264.7 cells, and both significantly inhibited osteoclastogenesis in calcitriol-stimulated mouse marrow. EVs from 4T1 cells treated with enoxacin and bis-enoxacin displayed small reductions in the amount of microRNA (miR)-146a-5p and let-7b-5p. In marked contrast, miR-214-3p, which has been shown to regulate bone remodeling, was increased 22-fold and 30-fold respectively. We conclude that enoxacin and bis-enoxacin trigger the release of EVs from 4T1 cancer cells that inhibit osteoclastogenesis.

## Introduction

Enoxacin is a fluoroquinolone antibiotic first introduced in the 1980s^1^. Removed from the market in the United States by its manufacturer, it is still used in many nations for the treatment of gastroenteritis, respiratory infections, gonorrhea and urinary tract infections^2^. Enoxacin emerged from two independent screens for bioactive agents as a possible therapeutic agent for cancer and bone disease. Shan *et al*. identified enoxacin as a stimulator of RNA interference and microRNA activity^3^. This result was soon confirmed by a second group^4^. General reduction in microRNAs in cancer cells may allow the expression of oncogenic proteins^5^. The ability of enoxacin to stimulate microRNA activity prompted studies testing it as an anti-cancer agent. Enoxacin inhibited cancer cell growth *in vitro*^6,7^, and *in vivo*^6^. A more recent study expanded on these results, demonstrating that enoxacin inhibited both proliferating cancer cells and cancer stem cells in Ewing’s Sarcoma, and that enoxacin acted synergistically with doxorubicin, a traditional anti-cancer agent, in the treatment of Ewing’s sarcoma in an animal model^8^. Tests of enoxacin, which crosses the blood-brain barrier, for the treatment for amyotrophic lateral sclerosis (ALS) have also provided promising results^9^.

Separately, Ostrov *et al*. identified enoxacin in a “virtual screen” for small molecules predicted to block binding between the B-subunit of vacuolar H^+^-ATPase (V-ATPase) and microfilaments^10^. This protein-protein interaction is important for bone resorption by osteoclasts^11^. Tests of enoxacin in biochemical assays and cell culture confirmed its ability to block binding between V-ATPase and microfilaments and to inhibit osteoclast formation and bone resorption^10,12,13^. Enoxacin was used to block titanium particle-induced osteolysis^14^, but its short half-life in circulation, the fact that it is an antibiotic, and its side effects, made it impractical to consider enoxacin as an agent to treat osteoporosis. To overcome these problems, a bisphosphonate ester of enoxacin (bis-enoxacin) that directed enoxacin to bone was synthesized^15,16^. Like enoxacin, bis-enoxacin blocked V-ATPase binding to microfilaments and bone resorption *in vitro* and orthodontic tooth movement and periodontal bone loss when delivered systemically in rats^15,17^. Surprisingly, it also reduced systemic oxidative stress induced by periodontal infections^18^. Very recent studies showed that bis-enoxacin protects bone strength in rats after overiectomy more effectively than zoledronate^19,20^. In addition to blocking bone mineral loss, it also altered the glycoprotein composition of bone, making it more resistant to fractures^19^.

Taken together, these data suggest that enoxacin and bis-enoxacin are a new type of therapeutic agent that may have distinct advantages over current therapeutics for the treatment of bone disease (bis-enoxacin) and cancer (enoxacin). Understanding the mechanisms that underlie the therapeutic effects is vital for the eventual use of these agents in the clinic.

Although enoxacin and bis-enoxacin completely blocked osteoclast formation *in vitro* at a concentration of 50 µM, over 100 µM enoxacin was required to inhibit cancer cell growth by 50% and 100 µM was used to demonstrate stimulation of microRNAs^6,7^. We hypothesized that these agents might have effects on cancer cells at lower concentrations that had not been detected, which might help account for their anti-cancer effects *in vivo*. In this report, we sought effects of low dose (50 µM) enoxacin and bis-enoxacin on 4T1 murine breast cancer cells, which have often been used to study the ability of breast cancer cells to invade bone^21–23^. Our data show that enoxacin and bis-enoxacin inhibit proliferation of 4T1 cells in a manner similar to various other cancer cells that have been tested *in vitro*^6,7^. Extracellular vesicles (EVs), which include exosomes and microvesicles, have emerged as important intercellular regulators in cancer invasion and in regulating bone remodeling^24^. We find that both enoxacin and bis-enoxacin alter the regulatory activity and composition of EVs released by 4T1 breast cancer cells at a lower concentration than that required to inhibit their proliferation.

## Results

### Enoxacin and Bis-enoxacin inhibit growth of 4T1 cancer cells only at high concentrations

**Figure 1:**
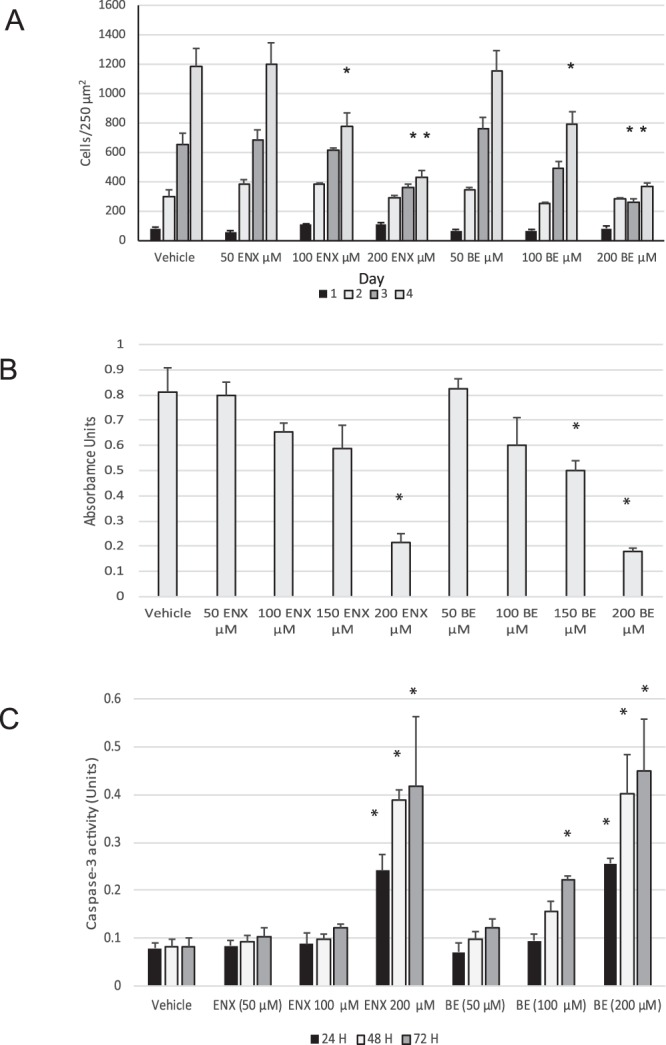
Enoxacin and bis-enoxacin, at a concentration of 50 µM, do not effect growth of 4T1 cancer cells or trigger apoptosis. (**A**) 4T1 murine breast cancer cells were plated at a density of 50 cells/250 µm^2^. After the cells had adhered on Day 1 and every 24 H subsequently, the cells in 3 randomly selected 250 µm^2^ squares (which was averaged) from at least 3 wells were counted by counters who were blinded to the treatment conditions. Data is presented +/− standard error. Asterisk indicates P < 0.05 determined by ANOVA and Student’s T test. (**B**) MTT assay was performed to examine proliferation of 4T1 cells. 2 × 10^4^ cells were plated in 96-well plates, treated with enoxacin, bis-enoxacin or vehicle as indicated and assayed after 2 days. The N for each condition as 8. Asterisk indicates P < 0.05 determined by ANOVA and Student’s T test. (**C**) 0.5 × 10^4^ cells/cm^2^ were plated in 24-well plates and treated as indicated. Caspase-3 activity was assayed at the times indicated. N for each condition was 4. Asterisk indicates P < 0.05 determined by ANOVA and Student’s *t* test.

Enoxacin inhibits the growth of various types of cancer cells *in vitro*, but requires concentrations of 100 µM or greater^6,7^. Enoxacin had not been tested on 4T1 cells. Enoxacin selectively inhibited osteoclast formation and bone resorption at much lower concentrations without inducing apoptosis^10,12^. Complete inhibition of osteoclast differentiation was achieved by 50 µM enoxacin or 50 µM bis-enoxacin and the IC_50_ for each was about 10 µM^12,15^. Suspecting that enoxacin and bis-enoxacin may have undetected effects on cancer cells at lower concentrations, we first examined concentration dependence. Consistent with reports on enoxacin’s effects on other types of cancer cells^6–8^, no changes in growth parameters were detected in the presence of 50 µM of either enoxacin or bis-enoxacin. At a concentration of 100 µM, 4T1 growth was inhibited by about 40% after 4 days (Fig. 1A). This level of inhibition was very similar to previous studies of the effects on enoxacin on other types of cancer cells^6–8^.

To further explore the effects of enoxacin, we used the MTT assay. As in cell counts, we detected no difference in proliferation at concentrations of 50 µM enoxacin and bis-enoxacin (Fig. 1B). We tested for apoptosis using a caspase-3 assay; there was no increase in caspase-3 activity at enoxacin and bis-enoxacin concentrations under 150 µM and apoptosis levels were modest even at high concentrations (Fig. 1C)

### Enoxacin and bis-enoxacin at 50 µM stimulated formation of GW/Processing (P) bodies but little increase in cellular levels of selected microRNAs was detected

**Figure 2:**
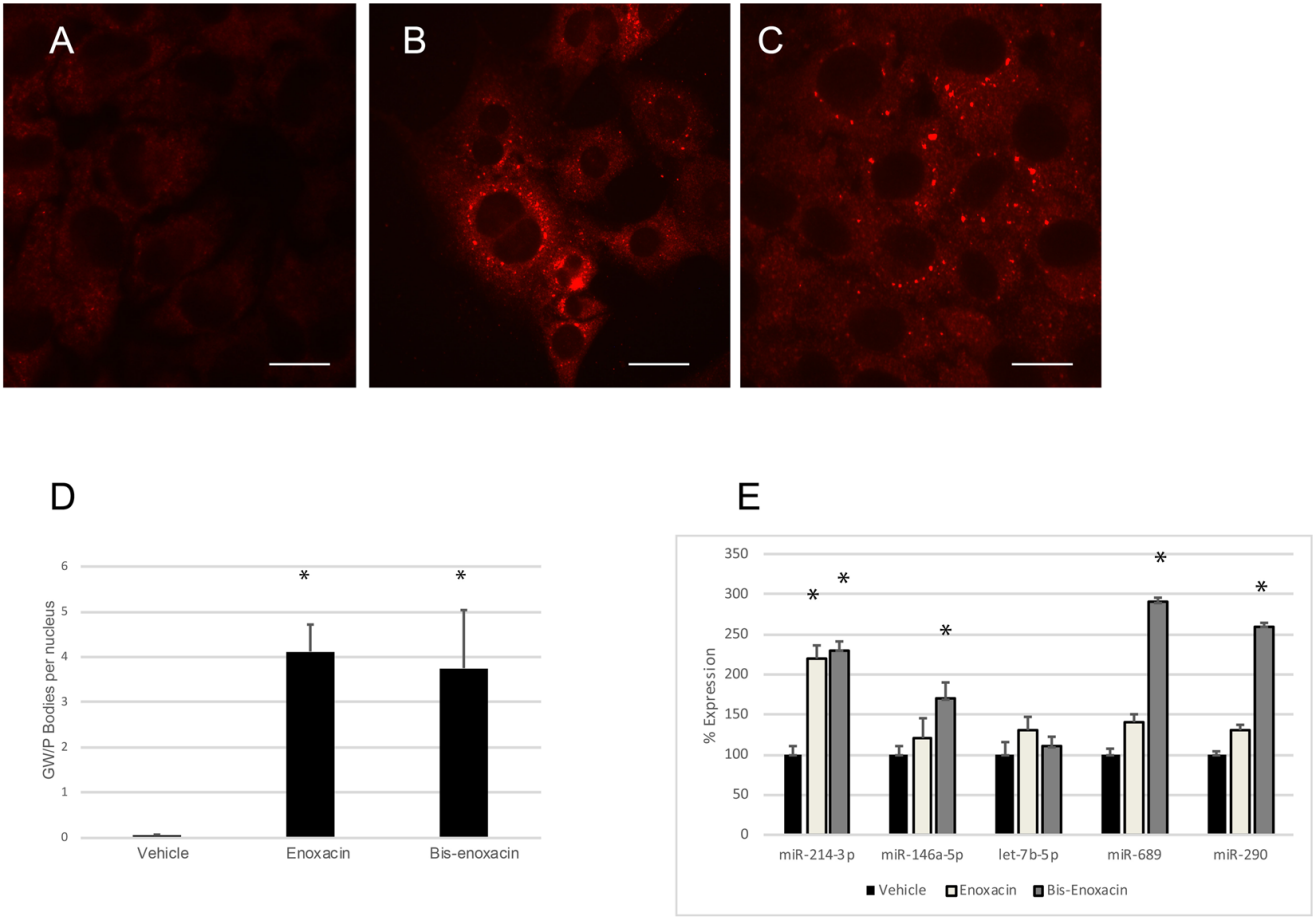
Enoxacin and bis-enoxacin at a concentration of 50 µM stimulate the formation of GW/P bodies but have little effect on microRNA levels. (**A**) Vehicle-treated control 4T1 cells stained with antibody that detects GW/P bodies. (**B**) Typical 4T1 cells from enoxacin-treated cultures stained with antibody that detects GW/P bodies. (**C**) Typical 4T1 cells from bis-enoxacin-treated cultures stained with antibody that detects GW/P bodies. (**D**) GW/P bodies were counted per nuclei, rather than by cell as significant numbers of multinuclear 4T1 cells were present in each condition. The scale bars are equal to 10 µm. Asterisk means p < 0.05 determined by Student’s T test. (**E**) Relative levels of cytosolic microRNAs have been determined by qPCR, through the ΔCT method and that p value has been calculated by Student’s t test. Asterisk indicates p < 0.05.

As a first test of whether low concentrations of enoxacin and bis-enoxacin affect cancer cells, we examined GW/P bodies, which are considered surrogate markers for microRNA-mediated repression of translation^25,26^. Enoxacin and bis-enoxacin, at 50 µM, stimulated significant increases in GW/P bodies (Fig. 2A–D).

To test whether microRNA levels increased, qPCR was performed to examine the relative levels of a panel of microRNAs that were selected based on published data. MiR-146a-5p, let-7b-5p and miR-214-3p were upregulated during osteoclast formation and were very abundant in osteoclasts. MiR-290 and miR-689 were abundant in precursors and downregulated as osteoclasts form^27–29^. For this reason, we considered them to likely be important regulators in osteoclasts. MiR-146a-5p and miR-214-3p have also been implicated in regulating bone remodeling^30–35^. Because enoxacin has been reported to generally stimulate microRNA levels and GW/P bodies are thought to be surrogate markers for microRNA increases, it was surprising that of the microRNAs examined, only miR-214-3p was significantly stimulated by enoxacin. Bis-enoxacin stimulated small, but significant increases, in cellular levels of miR-214-3p, miR-689 and miR-290 (Fig. 2E).

#### Enoxacin and bis-enoxacin induced the release of EVs by 4T1 cells that inhibit calcitriol-stimulated osteoclast formation

**Figure 3:**
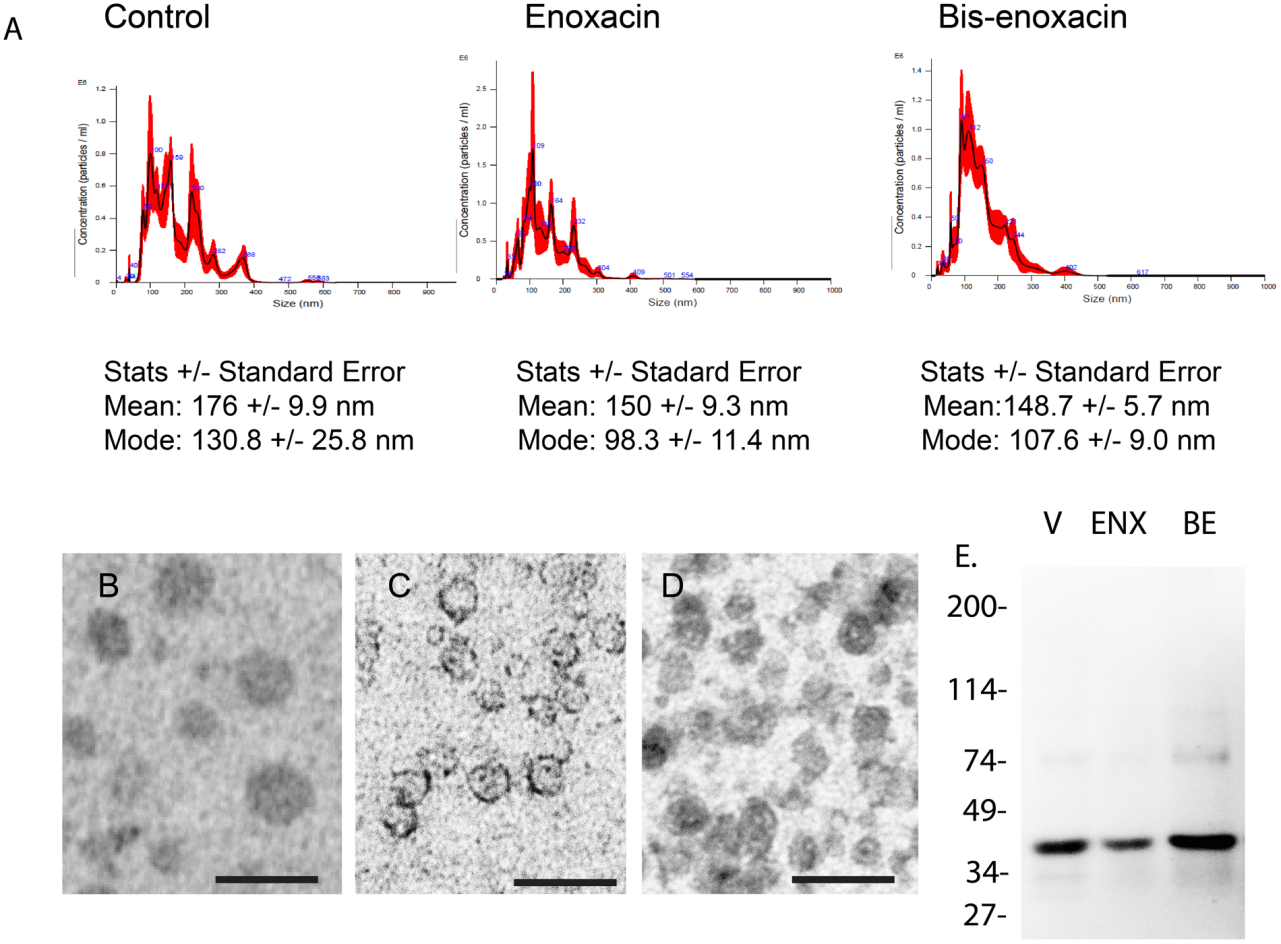
Enoxacin and bis-enoxacin reduce the size of EVs released by 4T1 cancer cells. (**A**) Typical nanoparticle tracking data. The mean and mode of EVs released from 4T1 cells treated with enoxacin or bis-enoxacin were significantly smaller than control EVs. (**B**) Imaging and quantitation by electron microscopy also suggested that there was a significant reduction in the size of EVs from enoxacin and bis-enoxacin treated 4T1 cells. Scale bars are equal to 100 nm. (**C**) Immunoblot of 100 µg of EVs from control, enoxacin and bis-enoxacin treated 4T1 cells separated on a 4–20% SDS-PAGE gradient gel and probed with an antibody to EpCAM, a marker for EVs from breast cancer cells.

In addition to being sites of microRNA-mediated translation repression, GW/P bodies have been proposed as sites where microRNAs are packaged into EVs^36^. EVs from 4T1 cells treated with vehicle, 50 µM enoxacin or 50 µM bis-enoxacin were examined by nanoparticle tracking. EVs from 4T1 cells treated with enoxacin and bis-enoxacin were significantly smaller (Fig. 3A). Positive/negative stain transmission electron microscopy confirmed that nanoparticles were EVs (Fig. 3B). An immunoblot was also performed showing that consistent with EVs from breast cancer cells^37^, EpCAM (CD326) were abundant in the isolated EVs (Fig. 3C).

**Figure 4:**
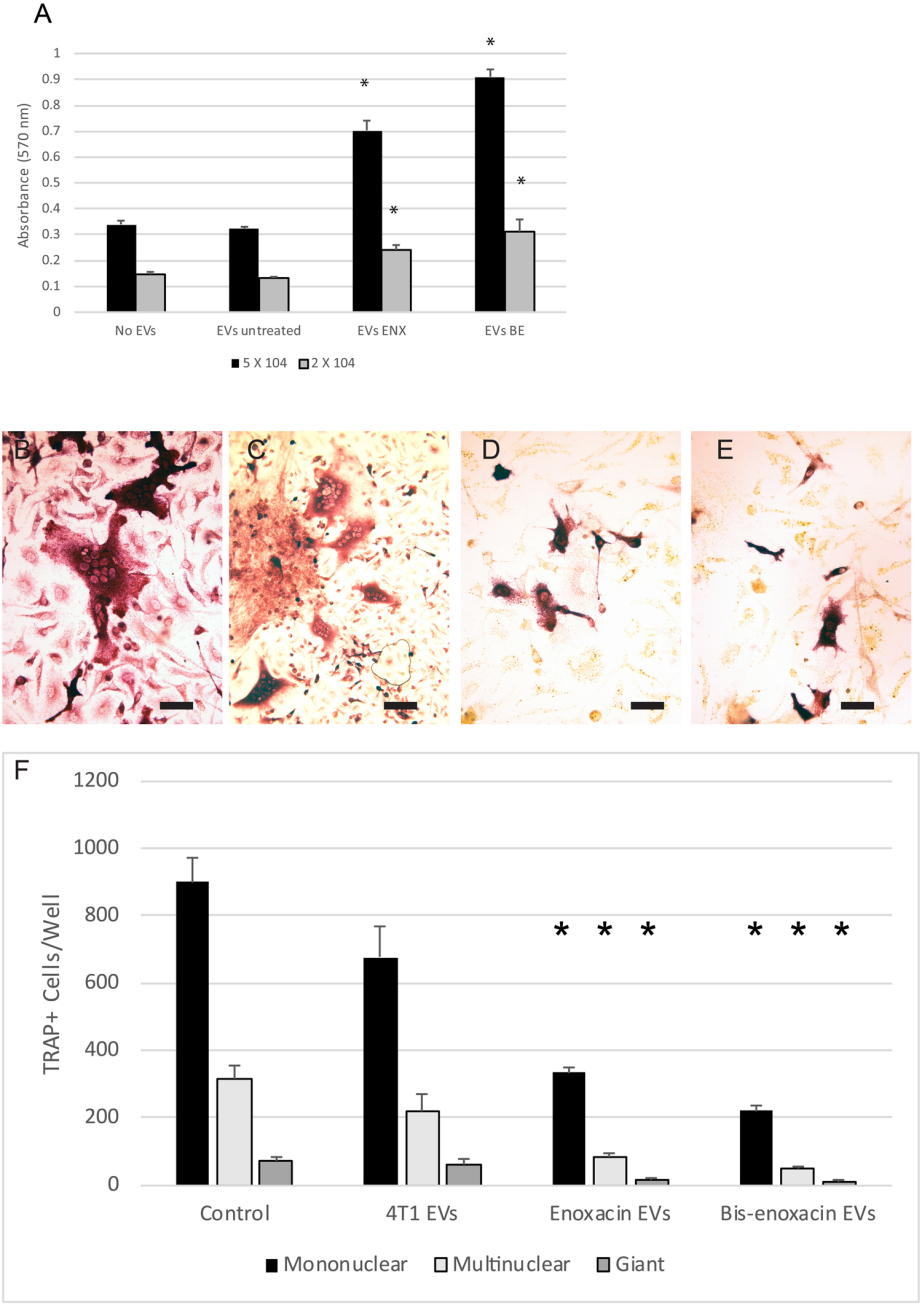
EVs from 4T1 cancer cells treated with 50 µM enoxacin or bis-enoxacin stimulate proliferation of RAW 264.7 cells, but inhibit osteoclast formation compared with EVs from untreated 4T1 cancer cells or no EV controls. (**A**) RAW 264.7 cells were plated at 2 × 10^4^ or 5 × 10^4^ cells per well and grown with no EVs or with 5 × 10^7^ EVs from vehicle treated 4T1 cells, 50 µM enoxacin-treated 4T1 cells or 50 µM bis-enoxacin treated 4T1 cells. The RAW 264.7 cells were grown for 2 days, then an MTT assay was performed. The N for each condition was 8. Asterisk indicates significantly-different from the no EV control at the same plating density by Student’s t test, P < 0.05. (**B**–**F**) Mouse marrow was cultured in the presence of calcitriol and 5 × 10^7^ EVs/mL with EV from vehicle-treated, from 4T1 cells or 4T1 cells from enoxacin and bis-enoxacin treated cells, or no EVs. Fresh EVs were added on the fourth day as the cells were fed. On day 6, the cells were fixed and stained for TRAP activity. (**B**) Representative photo from no EV control, (**C**) EVs from vehicle-treated control; (**D**) EVs from enoxacin-treated4T1 cells; (**E**) EVs from bis-enoxacin-treated 4T1 cells. The scale bars are equal to 10 µm. (**F**) Quantitative analysis of TRAP+ mononuclear, multinuclear (2–10 nuclei), and giant cells (≥10 nuclei). The N for each condition was 4 and the numbers of TRAP positive cells per well in the control cultures were mononuclear, 1133; multinuclear 487; and Giant, 94. Asterisks indicate different from control value, P < 0.05 by Student’s t test.

To test whether enoxacin or bis-enoxacin alter the regulatory activity of EVs from 4T1 cells, EVs were isolated from cells treated for 3 Days with vehicle, 50 µM of enoxacin or 50 µM bis-enoxacin. The EVs (5 × 10^7^ based on nanoparticle tracking) were then added to RAW 264.7 cells, a model for osteoclast precursors, which had been plated at a low and high cell density, and an MTT proliferation assay was performed. EVs from vehicle –treated 4T1 cells did not affect proliferation of RAW 264.7 cells. However, EVs from enoxacin and bis-enoxacin treated cells triggered significant increases in proliferation (Fig. 4A).

Calcitriol-stimulated mouse marrow were treated with 5 × 10^7^ control EVs or EVs from 4T1 cells treated with 50 µM enoxacin or bis-enoxacin on Days 1 and 4 of a 6-day culture period. The marrow cells were fixed and stained for tartrate-resistant acid phosphatase (TRAP) activity, a marker for osteoclasts, on day 6. EVs from vehicle treated 4T1 cells did not significantly alter osteoclast formation compared with no EV controls. EVs from enoxacin and bis-enoxacin-treated 4T1 cells significantly inhibited osteoclast formation compared with no EV controls as well as EVs from untreated 4T1 cells (Fig. 4B–F).

#### Enoxacin and bis-enoxacin increased microRNA-214-3p in EVs from 4T1 cells

**Figure 5:**
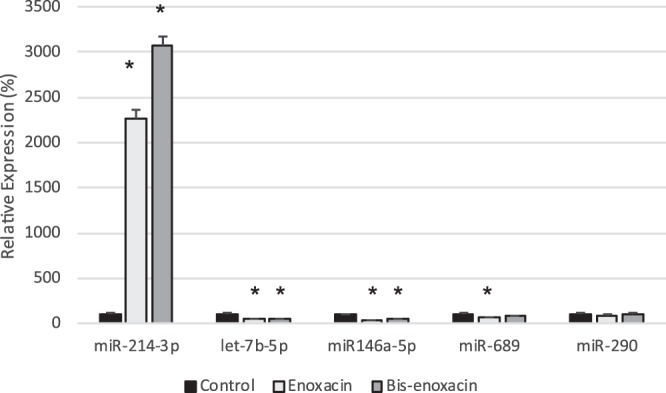
Enoxacin and bis-enoxacin stimulated large increases in miR-214-3p in EVs from 4T1 cells while reducing levels of other microRNAs. MicroRNAs were extracted from EVs for 4T1 cells and the amount of selected microRNAs (indicated) were quantitated by qPCR. Asterisk indicates p < 0.05 analyzed by the ΔCT method followed by Student’s t test.

MicroRNA (miR)-214-3p is involved in the regulation of osteoblasts by osteoclast EVs^33,38^. The relative levels of miR-214-3p and other selected microRNAa was determined by qPCR. Both enoxacin and bis-enoxacin stimulated large increases in miR-214-3p levels (22- and 30- fold), while significantly reducing the amount of miR-146a-5p and let-7b-5p detected in EVs (Fig. 5).

## Discussion

For the first time, we show that enoxacin and bis-enoxacin alter the regulatory activity of EVs released by a type of cancer cell at a relatively low concentration (50 µM). EVs from 4T1 murine breast cancer cells treated with enoxacin or bis-enoxacin, but not controls, increased proliferation by RAW 264.7 cells and strongly inhibited osteoclast formation in calcitriol-stimulated mouse marrow, which is a proven and widely-used cell culture model for the bone microenvironment. Enoxacin and bis-enoxacin treated 4T1 cells released EVs that were enriched 22-fold and 30-fold in miR-214-3p, while other microRNAs were reduced.

Why would EVs from enoxacin and bis-enoxacin-treated 4T1 cells stimulate proliferation of RAW 264.7 cells (a model for osteoclast precursors), but inhibit osteoclast formation in mouse marrow? In mouse marrow hematopoietic osteoclast precursors and mesenchymal osteoblast precursors carry on an intercellular communication dialog in response to calcitriol that leads to osteoclast formation. For this to occur in cell culture, the cells must be at the correct density and stage of differentiation. Disruption of any aspect of this differentiation process, like stimulating excess proliferation of osteoclast progenitors, could disrupt the overall process. At this point, we consider the crucial result to be the clear demonstration that treatment of 4T1 cells with low concentrations of enoxacin or bis-enoxacin alters the regulatory activities of the EVs that they release.

We found that enoxacin and bis-enoxacin inhibited proliferation of 4T1 cells with a concentration- dependence that was very similar to previous studies, which had examined various types of human cancer. These included colorectal, gastric, lung, breast, liver and pancreatic cancer, as well as leukemia and lymphoma^6,7^. Despite failing to affect proliferation, 50 µM of both enoxacin and bis-enoxacin stimulated GW/P body formation in 4T1 cells. Enoxacin at 50 µM significantly increased only one of five cellular microRNAs tested, miR-214-3p, while bis-enoxacin stimulated modest increases in the levels of miR-214-3p, miR-290 and miR-689. Our data show that enoxacin stimulates GW/P body formation in 4T1 cancer cells at concentrations where it does not function as a general stimulator of microRNA activity. This aligns with a very recent report that showed that although mouse embryonic fibroblasts lacking TAR RNA-binding protein 2 (TARP2), the proposed target of enoxacin^6^, were more resistant to growth inhibition by enoxacin^39^, changes in microRNAs stimulated by enoxacin were not influenced by the lack of TARP2. Taken together, these results suggest that the mechanism of enoxacin’s effects on cells may involve TARP2, but is likely more complex than general upregulation of cytosolic microRNAs. In addition to stimulating microRNAs, other proposed mechanisms for the observed therapeutic effects of enoxacin include, blocking interaction between V-ATPase B-subunit and microfilaments^12^, reduction in expression of lymphocyte cytosolic protein 1^40^, induction of alternative splicing of mdmX transcript and rescue of P53 activity^41^, ROS-activated DNA damage^42^, and suppression of the JNK-signaling pathway^14^.

Many recent studies suggest EVs released by cancer cells have the capacity to regulate cells in their microenvironment so that they accept the presence of the cancer cells and promote their survival^43–46^. For example, cancer-derived EVs containing transforming growth factor-β trigger cancer associated fibroblasts to differentiate into myofibroblasts and promote cancer growth and angiogenesis^47^. EVs from breast cancer cells have been shown to deliver microRNAs that alter glucose metabolism in the premetastatic niches to promote metastasis^48^. Integrins in EVs released by cancer cells have been implicated in their organotropism^49^. Transfer of integrins from tumor cells to non-tumor cells in EVs can trigger migration^50,51^. EVs have been reported to either promote immune response or be strongly immunosuppressive^52,53^. Pharmacological alterations of the regulatory activity of cancer EVs, as shown for enoxacin and bis-enoxacin, therefore may represent a novel type of anti-cancer therapeutic approach. Breast cancer cells are thought to stimulate the formation of osteoclasts and inappropriate bone resorption. This produces growth factors for the cancer cells and allows the cancer cells to have access to the bone^54^. The ability of enoxacin and bis-enoxacin to stimulate the release of EVs that inhibit osteoclast formation could be useful in the treatment of bone cancer.

Bis-enoxacin is a bone-targeted molecule that is an attractive candidate to be an agent to treat bone cancer. In addition to its intrinsic anti-resorptive effects, it also influences the EVs released by cancer cells so that they inhibit osteoclast formation. Changes in EV composition may also contribute to the effects of enoxacin and bis-enoxacin on bone remodeling. Recent studies support the idea that EVs from both osteoclasts and osteoblasts have roles in intercellular communication between osteoclasts and osteoblasts^33,38,55,56^. Transfer of microRNA-214-3p was recently implicated as a mechanism by which osteoclast EVs regulate osteoblasts^33,38^. The work of Li and colleagues provided evidence both *in vitro* and *in vivo* for a role for the transfer of miR-214-3p from osteoclasts to osteoblasts in EVs in the development of osteoporosis. Their data also correlated with human studies which showed increased levels of circulating miR-214-3p to be linked to osteoporosis. Our finding that miR-214-3p is greatly increased in cancer EVs provides a plausible mechanism by which the observed changes in the regulatory activities of EVs from 4T1 cells could be produced, although additional studies are required to test this hypothesis. Increased miR-214-3p in osteoclasts was identified as a mediator of bone cancer metastasis^34^, which suggests increasing its levels in cancer EVs may not be helpful. However, others showed miR-214-3p inhibited hepatocellular carcinoma proliferation^33^ and increased miR-214-3p enhanced the effects of cisplatin on squamous carcinoma cells^57^.

In summary, we find that enoxacin and bis-enoxacin alter 4T1 cancer cell-derived EVs so that they inhibit osteoclast formation in calcitriol-stimulated mouse marrow. Enoxacin and bis-enoxacin strongly enhanced the export of miR-214-3p from 4T1 cells in EVs while significantly reducing export of others. MiR-214-3p has been implicated as an important regulator of bone remodeling^33,35,58^. Finally, we suggest that bis-enoxacin represents a new potential agent for the treatment of bone cancer. By both inhibiting osteoclasts directly and triggering the release of EVs from cancer cells that inhibit osteoclast formation, it may prove useful for disrupting the ability of cancer cells to invade bone.

## Methods

### Reagents and Antibodies

Dulbecco’s minimum essential media (dMEM) and minimum essential media, α modification (αMEM) were obtained from Sigma/Aldrich Chemical CO (St. Louis, MO). The anti-EpCAM (D269-3) was obtained from MBL International (Woburn, MA, USA). Secondary antibodies were obtained from Sigma-Aldrich. ExoQuick TC and Exocet were obtained from Systems Biosciences (Mountain View, CA).

### Cell culture

4T1 murine breast cancer cells (kind gift of Gary Sahagian, Tufts University, Boston, MA) were grown in dMEM plus 10% fetal bovine serum (FBS) as described^59^. In some experiments the 4T1 cells were grown in dMEM plus 10% exosome-free fetal bovine serum (Systems Biosciences). Calcitriol-stimulated mouse marrow was prepared as described previously^60^. All protocols using of animals were approved by the University of Florida Institutional Animal Care and Usage Committee. All procedures were performed in accordance with guidelines of the National Institutes of Health of the United States. Cervical dislocation was performed to sacrifice Swiss-Webster mice. Femora and tibia were dissected from the mice, and marrow was removed by cutting both bone ends, inserting a syringe with a 25-gauge needle, and flushing the marrow using α-MEM plus 10% fetal bovine serum (α-MEM D10). Marrow was washed and plated at a density of 4 × 10^4^ cells/cm^2^ on 24-well plates for 5 d in α-MEM D10 with 10^−8^ M calcitriol plus EVs as noted. Cultures were fed on day 3 by replacing half the media per plate and adding fresh calcitriol. After 6 days in culture, osteoclasts were present. These were detected as giant cells that stained positive for tartrate-resistant acid phosphatase activity (TRAP; a marker for mouse osteoclasts). RAW 264.7 cells (ATCC), a cell line that serves as a model for macrophages and osteoclast precursors^11^, were grown in α-MEM D10.

### Growth Curves and Cell counting

Cells in three random locations (1000 µm × 1000 µm square) per well were counted by blinded counters. At least 3 wells per treatment group were counted at each time point.

### MTT assay

MTT proliferation assays were performed using a kit from Cayman Chemical (Ann Arbor, MI) according to the manufacturer’s directions. From 1 × 10^5^ to 5 × 10^5^ cells 4T1 or RAW 264.7 cells were seeded onto 96-well plates and cultured for 24 or 48 H. Absorbance was determined using a BioTek Synergy HT microplate reader (Winooski, VT) at 570 nm.

### Caspase-3 assay

Caspase-3 activity was assayed as described previously^12^. 4T1 cells were plated in 24-well plates at a density of 0.5 × 10^4^ cells/cm^2^ and treated with vehicle (DMSO), 50 μM enoxacin or 50 μM bis-enoxacin for 24, 48, and, 72 h. Caspase-3 assays were performed following the manufacturer’s instructions (catalog No. APT131, Millipore, Temecula, CA). At the end of each time point, the plate was centrifuged at 1200 rpm for 10 min. Cells were treated with 100 μl of chilled cell lysis buffer, and cell lysates were centrifuged at 1200 rpm for 10 min. Cell lysates were treated according to the manufacturer’s recommendations and the colorimetric reaction was quantified using a BioTek Synergy HT microplate reader (Winooski, VT) at 405 nm.

### Staining of GW/Processing (P) bodies

4T1 cells were plated on coverslips in 24-well plates. 5 × 10^4^ 4T1 cells were distributed to each well. The wells were treated with 50 μM enoxacin, 50 μM bis-enoxacin or vehicle (DMSO) control. After cells were treated for 24 hours, they were fixed in 2% formaldehyde in PBS for 10 min. The cells were then washed with PBS two times, for 10 minutes each, blocked in blocking buffer (PBS with a pH of 7.4 plus 1% bovine serum albumin (BSA)) for 60 minutes and probed with 200 μL of the human anti-GW bodies (GWBs) reference serum 18033, which reacts against GW182 and GE-1 at 4 °C overnight^61^. Then, the cells were washed two times in PBS for 10 minutes each, and secondary antibody, Alexa-Fluor-594 conjugated goat antibodies to human IgG was incubated for 60 minutes at room temperature in the dark. Then, the cells were rinsed two times in PBS for 10 minutes each. GW/P bodies were detected by epifluorescence microscopy. Nuclei were stained with 4′,6-diamidino-2-phenylindole (DAPI). The number of GW/Ps per cell, as well as the number of multinuclear cells, was quantitatively assessed using the computer program, ImageJ. Results are expressed as mean plus/minus standard error. Samples were compared by a Student’s *t* test using GraphPad software. P values < 0.05 were considered significant.

### Isolation of cancer EVs from cell culture and testing miRNA levels

Cells were grown in dMEM plus exosome-free media. The media was harvested and any debris and cells were removed by performing centrifugation at 5000 X g for 30 min. The cell-free culture media was then added to ExoQuick and incubated at 4 degrees Celsius overnight. The ExoQuick pellet was obtained by centrifugation at 5000 × g/30 min. EVs were then spun at 150,000 × g for 2 hours in an Airfuge (Beckman) in order to remove the solubilized ExoQuick. EVs were present in the pellet and then resuspended in sterile PBS. EVs were assessed by nanoparticle tracking. Quantitation of EVs was performed by using the Exocet kit (Systems Biosciences) following the manufacturer’s instructions or by nanoparticle tracking using a Nanosight NS-300 (Malvern). To determine mature miRNA expression, total RNAs were reverse-transcribed with High-Capacity cDNA Reverse Transcription Kit (Life Technologies) with a stem-loop RT primer designed based on the sequence of target genes (Applied Biosciences). Real-time PCR was performed using TaqMan® miRNA primers specific for miR-146a-5p (Mm000468 _m1), miR-290 (Mm000187_m1), miR-689 (Mm002635_m1), hsa-let-7b-5p (Mm000378_m1) and miR-214-3p (mmu481652_mir) with the StepOnePlus Real-Time PCR System (Applied Biosystems). Samples were normalized to small nuclear RNA U6 expression levels (part # 4427975). The experiment was analyzed statistically using *t* tests of the ΔCt values, or the relative expression levels of miRNAs were calculated using the 2^−ΔΔ*C*t^ method. *p* < 0.05 was considered significant.

### Size determination by Nanoparticle tracking

The diameter size and concentration of EV population was determined using a NanoSight NS-300 (Malvern). Samples were evaluated using different dilutions (1/10 to 1/50) in sterile-filtered PBS and the following parameters: camera at 30 frames per second, camera level at 16, temperature between 21–25 °C and video recording time at 60 s in order to estimate the concentration and size distribution of vesicles by light scattering and Brownian motion. Nanosight NTA Software analyzed raw data videos by triplicate.

### Transmission electron microscopy

EVs were visualized as described previously^55^. EV pellets were resuspended in 2% paraformaldehyde, placed on Parafilm, and Formvar-carbon–coated electron microscope (EM) grids were floated atop the EVs for 20 min. Grids were washed in PBS, transferred to 1% glutaraldehyde for 5 min, and then washed in deionized water 8 times. The grids were contrasted and embedded by placing them on a drop of uranyl-oxalate solution and transferred to methyl cellulose-uranyl acetate on ice for 10 min. The grids were blotted dry with filter paper and observed under an electron microscope. Electron microscopy was performed at the University of Florida, College of Medicine, Electron Microscopy Core Facility using a Hitachi (Tokyo, Japan) 7600 transmission electron microscope operated at 80 kV.

### Immunoblotting

EVs isolated from 4T1 cells treated with vehicle, enoxacin or bis-enoxacin were extracted by solubilizing the pellet in Laemmli buffer (BioRad, Hercules, CA) followed by boiling for 5 min. Samples were then subjected to sodium dodecyl sulfate polyacrylamide gel electrophoresis on 4–20% gradient gels and transferred to nitrocellulose. Blots were probed with Anti-EpCAM (D269-3) obtained from MBL International (Woburn, MA, USA).

### Detection of osteoclasts

Osteoclasts were detected by staining for tartrate resistant acid phosphatase (TRAP) activity as described previously^60^. Cells were counted by volunteers who were blinded to the treatment conditions and who had been calibrated. We documented mononuclear, multinuclear (2–10 nuclei) and Giant (>10 nuclei) cells that were positive for TRAP activity as described previously^60^

### Statistics

Results are expressed as mean plus/minus Standard Error. Samples were compared by One-Way ANOVA and Student’s *t* test using the program GraphPad Prism 5 (GraphPad Software, La Jolla, CA). P values < 0.05 were considered significant. Nanoparticle tracking was analyzed by ANOVA with Tukey’s modification.

## Data Availability

The datasets generated during and/or analysed during the current study are available from the corresponding author on reasonable request.
